# Patients' Experiences of Living with Atrial Fibrillation: A Mixed Methods Study

**DOI:** 10.1155/2019/6590358

**Published:** 2019-12-03

**Authors:** Marie Stridsman, Anna Strömberg, Jeroen Hendriks, Ulla Walfridsson

**Affiliations:** ^1^Feelgood Företagshälsa, Linköping, Sweden; ^2^Department of Medical and Health Sciences, Division of Nursing, Linköping University, Linköping, Sweden; ^3^Department of Cardiology, Linköping University Hospital, Linköping, Sweden; ^4^Centre for Heart Rhythm Disorders, University of Adelaide, South Australian Health & Medical Research Institute and Royal Adelaide Hospital, Adelaide, Australia

## Abstract

**Introduction:**

Awareness of epidemiological and clinical consequences of atrial fibrillation (AF) has increased, as have disease-related costs. Less attention has been paid to patient-related issues, such as understanding how symptoms, different therapies, and lifestyle adjustments affect daily life. We aimed to describe patients' experiences of living with AF.

**Methods:**

The study design used a parallel convergent mixed methods approach. Patients with AF were included in the SMURF study and referred for catheter ablation. Patients completed questionnaires on symptoms, health-related quality of life, depression, anxiety, and perceived control and were interviewed. The datasets were analysed separately using inductive content analysis and descriptive statistics. Data were merged to obtain a final interpretation.

**Results:**

Nineteen patients were interviewed and 18 completed questionnaires. Twelve of the patients were male, mean age 60 years (45–75 years). Inductive qualitative analysis revealed three categories: (i) symptoms and concerns limiting life, (ii) dimensions of worries, and (iii) strategies for management. The most common symptoms were tiredness, weakness/fatigue, and breathlessness during activities, and the most pronounced negative impacts on health-related quality of life (HRQOL) were physically related, shown in the ASTA questionnaire. The most negative SF-36 scores were found in role limitations due to physical health problems and vitality. HADS revealed five patients with some degree of anxiety and four with some degree of depression. Patients had lower scores on perceived control than perceived helplessness in CAS. Patients' perceived control was higher than their families', and families experienced more helplessness.

**Conclusions:**

The mixed methods design deepens our understanding of challenges faced by patients. Patients experienced a limited ability to perform activities of daily living due to AF which created different kinds of worries that encouraged the use of various strategies to manage their lives. Healthcare providers need to be aware that relationships between patients and their relatives can change, and therefore they need to be supported and integrated into the care system.

## 1. Introduction

The treatment of patients with atrial fibrillation (AF) is mainly driven by symptomatology. It is well known that some patients have a disabling symptom burden, while others barely experience any symptoms [1]. Difficulties with activities of daily living are not only a problem for the patient but also for the relatives, where some patients place self-imposed restrictions on their behaviour in fear of AF recurrences and thus limit everyone's activities of daily living [2, 3]. Evaluating treatment outcomes cannot be sufficiently made by objective measurements alone. There can be discrepancies between ECG results and how the patient feels [4]. In person-centred care, healthcare providers need to consider patients' perspectives [5, 6]. Interviewing patients is one way to explore disease-specific concerns, but it is time consuming and difficult to implement in clinical practice [7, 8]. A more applicable approach is to use validated [9–15] and disease-specific questionnaires, developed for the targeted patient population [16, 17].

Management of AF should be comprehensive, including the treatment of arrhythmia, appropriate prescription for anticoagulation, and treatment of underlying cardiovascular comorbidities, and also should focus on risk factors and lifestyle management [18]. Current guidelines recommend that the choice of management strategy should be guided by the symptomatic status of the patient [1]. While awareness of epidemiology and clinical consequences, as well as AF-related costs, have increased over the past few decades, less attention has been paid to patient-related issues. Patients report limitations in both their professional lives and leisure activities [19]. Patients with AF may feel more uncertain with regard to their diagnosis, treatment, and prognosis than those with other illnesses [20]. It is reported that patients felt uninformed and unsupported in their care process to understand, deal with, and respond to their symptoms. Consequently, emotional stress burden is high for these patients [7, 20]. It is vital to understand the impact of symptoms and how patients are affected in their daily lives. Guidelines recommend engaging with patients and actively involving them in their care process, providing education and ensuring that treatment is not only based on guidelines but also fits the needs, preferences, and values of patients [1].

Not knowing when the next AF episode will occur leads to uncertainty which can negatively impact daily life, both for patients and their relatives [5, 7]. Patients often experience palpitations, breathlessness during activity, tiredness, weakness, and fatigue. Some symptoms can be very disabling and greatly influence daily living [1]. Patients with AF experience poorer health-related quality of life (HRQOL) compared with the general population and patients with other heart conditions [21]. Symptoms of anxiety and depression are highly prevalent in patients with AF, which further reduces HRQOL for patients and their spouses [22].

Most studies that explore patients' experiences of living with AF are either based on interviews or patient-reported outcomes via questionnaires [7, 8, 21, 23]. The aim of the current study was to describe patients' experiences of living with AF, using a mixed methods approach to confirm and deepen our understanding of patients' situations.

## 2. Methods

### 2.1. Study Design and Participants

This study has a parallel convergent mixed methods design. The qualitative data were the primary set of data that guided the sample size of the study [24, 25]. Data were collected using questionnaires and semistructured interviews. The datasets were first analysed separately and then merged into a final interpretation [24]. The symptom burden, metabolic profile, ultrasound findings, rhythm, activation of natriuretic peptides, haemodynamic and diastolic function, and HRQOL in AF (SMURF) study included 192 patients with AF undergoing their first radiofrequency ablation (RFA), from which 19 patients were selected for this mixed methods substudy. The first eight interviews were performed according to a convenient sampling. After a discussion in the author group, patients were purposefully selected to include some more women and also some younger patients. Patients were recruited from a university hospital in southeastern Sweden. Inclusion criteria were persistent or paroxysmal AF, age ≥18 years, referral for first RFA, and the ability to fill out the questionnaires independently. More details are presented elsewhere [26, 27]. Data were collected between August 2011 and December 2012. The sample mirrors a patient population referred for RFA regarding age, sex, symptom burden, and comorbidities.

### 2.2. Ethical Considerations

The study protocol was approved by the Regional Ethical Review Board at the Faculty of Health Sciences, Linköping, Sweden (DNR 2011/40-31, 2012/226-32). The participants received oral and written study information and gave written informed consent. The study complies with the Declaration of Helsinki [28].

### 2.3. Measures: Questionnaires

#### 2.3.1. Arrhythmia-Specific Questionnaire in Tachycardia and Arrhythmia (ASTA)

The Arrhythmia-Specific questionnaire in Tachycardia and Arrhythmia (ASTA) is divided into three parts. ASTA part I evaluates the patient's latest episode of arrhythmia and current medication. Part II assesses symptom burden with a 9-item symptom scale and a 4-point response scale. There are questions concerning the frequency, average and longest duration of arrhythmia, and patients' experiences of near syncope and syncope in connection with arrhythmia. Part III assesses HRQOL with a 13-item scale and the same 4-point response scale as for the symptom scale. The ASTA HRQOL scale can be divided into physical and mental subscales. Higher scores reflect a higher symptom burden and a worse effect on HRQOL due to arrhythmia [16, 17]. Scale scores can also be calculated, ranging from 0 to 100 where higher scores reflect a higher symptom burden and a worse effect on HRQOL due to arrhythmia.

#### 2.3.2. Medical Outcomes Study (MOS) 36-Item Short-Form Health Survey (SF-36)

The 36-item short-form health survey (SF-36) is a generic questionnaire assessing an individual's physical and mental health. It comprises 35 items grouped into eight scales and one question concerning changes in health not covered in the eight scales. The scales represent physical functioning (PF), role limitations due to physical health problems (RP), bodily pain (BP), general health (GH), vitality (VT; energy/fatigue), social functioning (SF), role limitations due to emotional problems (RE), and mental health (MH; psychological distress and psychological well-being). The scales can be summarised as physical and mental component summary scores (PCS and MCS). Scores range from 0 (worst possible health) to 100 (best possible health). Scoring of the SF-36 data was carried out as described by Ware and colleagues [10, 29]. SF-36 has been used in research, including studies of patients with arrhythmias [6, 21].

#### 2.3.3. Hospital Anxiety and Depression Scale (HADS)

The domain-specific questionnaire Hospital Anxiety and Depression Scale (HADS) is used to evaluate symptoms of anxiety and depression. Seven questions assess anxiety (HADS-A) and the remaining seven assess depression (HADS-D). Responses are scored on a scale of 0 to 3, with higher scores denoting more psychological distress. Scores for each subscale (anxiety and depression) range from 0 to a maximum score of 21 [12, 13].

#### 2.3.4. Control Attitudes Scale (CAS)

The Control Attitudes Scale (CAS) measures perceived control among patients with cardiac disease [14, 15]. The scale consists of four items: two about perceived control and two about helplessness. Furthermore, two of the items reflect the patient's own perceptions and two reflect the patient's perception of family members' perceived control [24]. Patients rate each item on a 7-point scale, from “Not at all” to “Very much.” The total score varies between 4 and 28, where higher scores indicate greater perceived control.

### 2.4. Measures: Interviews

Semistructured interviews were performed. Three of the authors (MS, AS, and UW) created an interview guide: (1) “Tell me about your experiences of living with AF.” (2) “Tell me about your symptoms and problems related to AF.” (3) “Is there anything that provokes AF episodes?” (4) “Is there anything that helps you to cope with AF during and between the AF periods?” (5) “Are your relatives or friends affected by your AF?” The questions were pilot tested and retained without any changes. The interviews were conducted by one author (MS) at the clinic where patients underwent ablation, 1–4 days before the treatment.

Each interview lasted 25 to 50 minutes and was audio-recorded and transcribed verbatim. After transcription, the interviews were read at the same time as the first author listened to the audio recordings. Data collection continued until the data became repetitive and redundant.

The first eight interviews were performed according to convenience sampling. After a discussion in the author group, patients were purposefully selected to include more females and also some younger patients. We aimed to include a broad range of patients representing diverse aspects of how living with AF affects daily life. All patients who were approached for the interviews agreed to participate.

### 2.5. Data Analysis

#### 2.5.1. Quantitative Analysis

Frequencies and median values with 25th to 75th percentiles were used to describe patient characteristics and questionnaire outcomes. First, we compared the quantitative analysis of ASTA, SF-36, HADS, and CAS. Statistical analysis was conducted using SPSS 22 for Windows (SPSS, Inc., Chicago, IL, United States).

#### 2.5.2. Qualitative Inductive Content Analysis

Qualitative inductive conventional content analysis was performed with Hsieh and Shannon's steps to guide the analysis [30]. Then, we read the transcripts to obtain an overall sense of the content. Three authors independently highlighted words and sentences of interest (meaning units) in the transcripts and thereafter discussed the highlighted text to obtain a consensus about the main themes of responders' experiences. Next, we organised the data with open coding. Single words and sentences that captured the essence of the aims were highlighted.

The data were coded using NVivo9. Codes were grouped into higher-order categories, and those belonging together were merged into new codes [31]. Categories were named, and their contents were described [30]. Internal logic and consistency were verified with authentic quotations from the text [32, 33]. A coding scheme for each category was developed [34]. The consistency of the coding was checked by three authors, and the codes were discussed until consensus was reached. The codes and categories were compared and contrasted against the original text throughout the analysis. The process involved reading back and forth between the text and units to ensure stringent and trustworthy analysis.

#### 2.5.3. Mixed Methods Analysis

Finally, we compared the quantitative data obtained from the questionnaires with the three categories derived from the inductive qualitative analysis (i.e., interviews). The categories were compared with similar domains within the questionnaires. Analysis and interpretations were made to explore if the results were comparable and convergent, whether the combination of qualitative and quantitative data expanded the understanding of patients' experiences of living with AF, or if the results were inconsistent [24].

## 3. Results

### 3.1. Patients

A total of 19 patients were interviewed, and 18 of them completed the questionnaires. Twelve of the substudy patients were male (63%), and the mean age was 60 years (range 45–75 years); 14 patients (74%) had paroxysmal AF, and the most common comorbidities were hypertension, heart failure, and diabetes (Table 1).

### 3.2. Quantitative Findings

In the ASTA questionnaire, the most commonly reported symptoms were tiredness, weakness/fatigue, and breathlessness during activities. Ten patients had been close to fainting, and four fainted in connection with AF. Most pronounced negative impacts on HRQOL were impaired physical ability, deteriorated living situation, and an inability to carry out daily activities and to plan leisure activities. Ten patients felt that their sexuality was negatively affected by AF (Table 2). There were no missing data in the ASTA questionnaire.

The best health scores measured by SF-36 were for bodily pain, mental health, and physical functioning. The lowest health scores were found in role limitations due to physical health problems and vitality. Two patients had one missing item each.

HADS revealed that two patients scored a mild degree of anxiety, two a moderate degree, and one had severe anxiety. One patient scored a mild degree of depression, and three patients scored a moderate degree (Table 2). There were no missing data in the HADS questionnaire.

Patients had lower scores on perceived control (mean 6.3 ± 2.7) than perceived helplessness (mean 8.0 ± 3.4) according to CAS results. Patients reported their perceived control to be higher than their families' degree of control. They also scored that their families experienced more helplessness than they did themselves. There were two patients with missing data on CAS.

### 3.3. Qualitative Findings

The inductive qualitative analysis revealed three categories: symptoms and concerns limiting life, dimensions of worries, and strategies for management.

### 3.4. Symptoms and Concerns Limiting Life

Symptoms varied greatly. Some patients were less symptomatic, while others reported unpleasant palpitations along with fast irregular heartbeat and dizziness during AF and reported feeling close to fainting.

They felt weakness, tiredness, and breathlessness during activities. Patients experienced physical limitations during and after episodes of AF with reduced capacity to perform activities. Fatigue was described as “a constant companion.” Not being physically active resulted in weight gain for some patients. Patients described that they needed to rest more, avoided tiring activities, and had to reduce their pace of activities.

“You do not clean up so much, have no energy, and do not bother about gardening.”

Patients expressed feeling that they were in an unusual situation by being dependent on others for housework, gardening, and driving longer distances. Due to their lack of energy, they were unable to work and withdrew from other people and social activities.

“I withdrew, wanted to be alone, and could not cope with being around people.”

Some male patients expressed negative effects on sexual function. They experienced loss of sensations and reported impotence, which negatively affected their self-esteem. Patients related these effects to antiarrhythmic medications.

The limitations were also mental. Not being able to perform usual activities during AF caused restlessness accompanied with frustration. Feeling tired affected the patients' mood. They became increasingly irritated, which had a negative effect on their social lives.

“You become like a child, a bit fussy when you are tired.”

They lost interest in activities and things that they once considered important. One patient described that she did not bother about her appearance anymore. Due to daytime sleepiness, patients felt that they had lost mental capacity.

“Mentally you are not sharp and you are doing the wrong things, you do not really “get” things.”

It was more common to forget things and to have difficulties in focusing and concentrating after the diagnosis. One patient described the inability to concentrate on something else other than the symptoms of AF:

“When you only focus on atrial fibrillation, you get nothing else done.”

### 3.5. Dimensions of Worries

Never knowing when the next episode might occur led to an insecure living situation with a constant feeling of worry for both patients and families. It was common to be less active and to avoid planning for leisure activities, for example, travelling long distances. Patients did not want to cancel arrangements, said no to invitations, and felt that they became “isolated.”

*“You become limited, restricted*, *and do not participate, you say no and do not do things you would like to do.”*

When they planned activities, they felt insecure about the coming event and they described how they felt nervous and looked for symptoms of AF the whole time.

“You worry about practical aspects of having a relapse of AF, how to get home and where to leave the kids.”

Palpitations could be annoyingly painful, with tension and pressure in the chest or throat. It often caused worries and sometimes sleeping problems. Patients stated that the first experience of AF was very frightening, especially before being diagnosed and receiving an explanation about what they experienced.

“The first time I had it, I was really worried. I hardly dared to fall asleep because I thought I might never wake up again.”

Repeated AF episodes caused a lot of distress. Patients worried about long-term effects and feared the infliction of serious damage to the heart. They were afraid of cardiac arrest, stroke, and experiencing impaired physical capacity. Some expressed a fear of dying.

“You have only one heart.”

Other worries were fear of adverse drug reactions and fear of complications in connection with RFA. The extra healthcare controls and blood samples, which were sometimes needed, increased levels of anxiety.

“Oh my God, am I going to take those damn medicines for the rest of my life? It could certainly not be good at all.”

It was difficult for respondents to reconcile themselves to being patients, and it was common to feel that it was “unfair” and “scary” to be young and to have AF.

Patients had to manage worries concerning their family, and this could worsen the situation.

“My wife is very worried, but she does not dare to show it because she knows that it makes me worried.”

Some patients avoided mentioning recurrences to not worry family members. Family members' behaviour could cause annoyance. Patients could get irritated due to repeated questions such as “How do you feel” or due to relatives taking their pulse.

### 3.6. Strategies for Management

A common strategy was to seek knowledge from healthcare providers, other patients, and the Internet. Sharing common problems with other patients provided valuable support and reduced feelings of being alone with the disease. The amount of information patients wanted and actually received varied. While some struggled to get information, others established good contact with healthcare providers and found them informative. Written information with illustrations was appreciated.

“Knowledge helps, and makes it easier to get to know myself.”

Most patients tried to manage AF by identifying possible factors that provoke arrhythmias. They spent much time thinking about what could start a new episode, what they had done, what they had been eating, and if they had been in a particular mood before relapse. Some expressed nearly an obsession and others found it frustrating not being able to find what triggered the onset.

“I tried to guess so much, tried until I got almost crazy about it.”

Factors found were, for example, certain fatty and spicy foods, eating big meals late in the evening, drinking alcohol or coffee, physical exercise, sleep deprivation, and mental stress. It was also common for AF episodes to occur during rest or at night.

The methods to manage relapses varied. Some tried to breathe deeply and calmly, others rested. While some performed heart rate accelerating activities to interrupt the attack or distract themselves from symptoms, most patients preferred stillness in a quiet place.

“The safety of home, when you do not feel well, is probably the most important thing of all.”

Coping mechanisms reported were finding problem-focused coping strategies, getting support from family and friends, and stopping unpleasant emotions. When people have a chronic disease like AF, they cannot afford to focus on the problem, as it will never disappear. Patients reported that the best they can do is to stop unpleasant emotions and to behave as if the problem did not exist.

Many patients said that they tried to live as normally as possible, as they had before their first episode of AF. They reported that they had not changed their lifestyle because they did not want their life to be limited.

“I want it to affect me as little as possible. I do not want to be ruled by my AF.”

Some tried to think positively and comforted themselves with peaceful thoughts while others sought healthcare assistance.

Patients were at different stages regarding their acceptance of an AF diagnosis. There were those who did not want to be characterised as a patient. Over time, they learned how to cope with the limitations and adhered to the boundaries of exercise and activities.

“Avoiding, yes of course, I have been suffering from it for so long now, it has become a part of me, and yes, I know I can manage it.”

### 3.7. Mixed Methods Results

#### 3.7.1. Symptoms and Concerns Limiting Life

Symptoms such as tiredness, weakness, and fatigue were convergent and commonly reported both in the ASTA questionnaire and in the interviews. The negative impact of arrhythmias on HRQOL was mostly physically related, which became obvious in the ASTA questionnaire and was demonstrated with the lowest scores in the scales: role limitations due to physical health problems and vitality in the SF-36 questionnaire. Interviews confirmed difficulties about not being able to perform physical activities. The mixed methods design made it possible for male patients who reported a negative influence on their sexual life to explain what they believed to be the reason for their problems.

#### 3.7.2. Dimensions of Worries

Most of the mental concerns were convergent and detected in both the datasets. The influence of AF on patients' ability to concentrate and their mood were assessed in ASTA and SF-36's role emotional and mental health scales. The interviews confirmed and deepened our understanding that AF has a negative mental impact and leads to experiences of frustration and restlessness. More than half of the patients reported anxiety related to AF in the ASTA questionnaire. In the interviews, patients described different experiences of dealing with worries, with several examples of fear of palpitations, and negative effects of AF and its treatment. Worry from relatives was often taken into consideration according to both the interviews and CAS. This affected both the patients and the relatives, which, in turn, influenced their relations. However, most of the patients recorded a normal HADS score in relation to anxiety and depression.

#### 3.7.3. Strategies for Management

The factors that might provoke AF recurrences were common across ASTA and the interviews, but the activities performed to manage relapses were elaborated in the interviews. Patients used different strategies to manage relapses, and the interviews expanded information about how patients were seeking knowledge about their disease to avoid and manage relapses and were trying to live a life as normally as possible. Many reported that information and support from healthcare providers helped them to manage AF.

**Figure 1:**
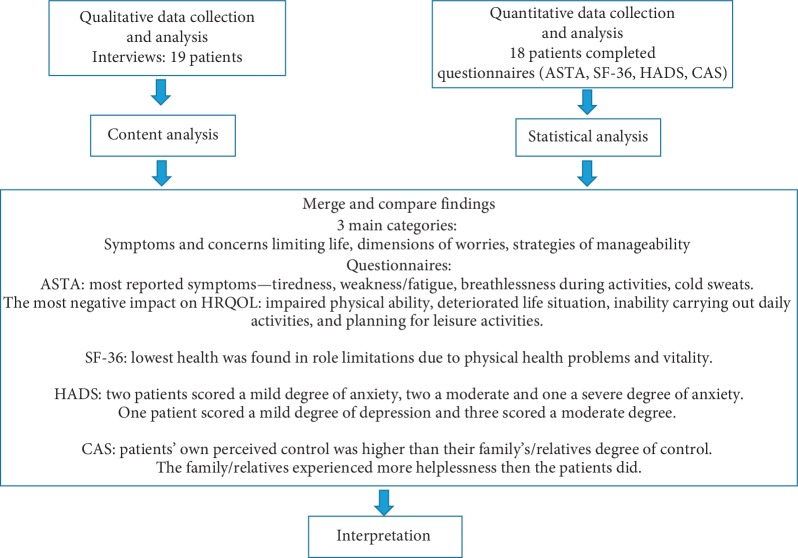
Description of mixed method design used to describe patients' experiences of living with atrial fibrillation. ASTA = Arrhythmia-Specific Questionnaire in Tachycardia and Arrhythmia; SF-36 = Short-Form health survey; HADS = Hospital Anxiety and Depression Scale; CAS = Control Attitudes Scale; HRQOL = health-related quality of life.

The challenging situations faced by patients and relatives, with feelings of helplessness and low control, were also expressed in the interviews and CAS (Figure 1).

## 4. Discussion

This study aimed to describe patients' experiences of living with AF using a mixed methods approach to achieve a deepened understanding of the challenges that patients with AF and their relatives deal with in daily life. Inductive qualitative analysis revealed three main categories: symptoms and concerns limiting life, dimensions of worries, and strategies for management. The symptoms hindering patients' activities of daily living and the most negative HRQOL concerns were physically related. Role-physical scores on the SF-36 were consistently the lowest SF-36 scores, followed by vitality.

It is well known that patients with AF have a HRQOL which is negatively influenced by their arrhythmias [21]. In a previous study, we investigated HRQOL in cardiac patients with supraventricular tachycardia, using the SF-36. This was a young and otherwise healthy population. Interestingly, the findings of the current study demonstrated similar results, meaning higher scores on PF, while scores on RP and VT were impaired [35]. It is shown that patients more often report pronounced physical impairment. Factors such as older age, female sex, and more severe symptoms lead to a decreased HRQOL, particularly in physically related domains [23, 36].

### 4.1. Symptoms and Concerns Limiting Life

Notably, more than half of the patients reported having disabling symptoms such as being close to fainting and some had even fainted. This confirms results of an earlier study, where being close to fainting was shown to precede self-imposed restrictions in common daily activities, for instance, driving [3]. Physical activities including training programmes are often recommended for patients with coronary heart disease and heart failure. The negative impact of AF on physical capability can be problematic, and it is important to encourage a healthy lifestyle. It is known that an essential component for long-term efficacy of catheter ablation is lifestyle management including weight reduction to improve multiple risk factors and ease the symptom burden [37–39].

Experiences of sexual problems in patients with cardiovascular diseases are common [40]. The negative consequences of AF on patients' sexual lives are less frequently discussed. In the present study, some patients described problems with reduced sensation and impotence and related these problems to medication side effects. This issue is important for patients' self-esteem and needs to be addressed in the care of patients with AF.

### 4.2. Dimensions of Worries

Our results demonstrate how stressful it can be to live with AF. The vulnerable situation and uncertainty for patients with AF have been described previously in patients living with supraventricular tachycardia [41] and in those carrying an ICD [42] who report feelings of social isolation. When patients withdraw from social activities due to AF symptoms, it negatively affects relatives. Patients have reported relatives' worries as being even higher than their own [42]. Not surprisingly, it has been reported that the well-being of relatives is affected by worry for their relatives with AF and that relatives had to sacrifice their own needs [2]. We found patients scoring higher than their relatives on perceived control, and relatives experienced more helplessness than patients did. There were patients who avoided telling relatives and friends about reoccurrences of arrhythmia and who tried to hide episodes of arrhythmia in order to limit the worry of others [41]. The phenomenon where relatives' behaviour could cause annoyance was also found in our study.

Symptoms of depression and anxiety were described in this patient group and are associated with increased symptom severity [26, 43, 44]. Patients in this study scored within the normal range for domains of anxiety and depression in HADS. The ASTA demonstrated that more than half of the patients reported worries/anxiety related to AF. This has to be taken into consideration in the care planning for these patients [8].

### 4.3. Strategies for Management

Patients should be advised and supported to seek advice from their physician and should also be directed to contact one of the AF patient associations for more general advice and support.

It is crucial that patients receive continuous and comprehensive education regarding their condition and treatment in order to empower them to undertake self-management, to learn how to prevent or limit factors that trigger AF, to find out what to do during episodes of AF, and to know when to report episodes to the hospital. A recent randomised controlled study demonstrated that structured education, tailored to the individual patient, significantly reduced unplanned hospitalisation [45]. Novel models of care in terms of integrated care approaches and specialised clinics, in which there is a clear focus on patient education, have demonstrated a significant reduction in clinical outcomes such as cardiovascular hospitalisation and mortality [46].

## 5. Methodological Strengths and Limitations

The aim of the present study was to explore patients' situations related to AF using a combination of interviews and questionnaires to gain a comprehensive picture of the influence of AF on their daily living. The mixed methods design contributed to the broadening of this insight and, at the same time, is a methodological approach that is novel in this patient population. Earlier publications in this field were either designed to be qualitative, with interviews, or took a quantitative approach, for example, using questionnaires [7, 8].

The quantitative data from this study should be interpreted carefully, given that the sample size was small. These data were used to triangulate the qualitative findings. As the study population consisted of symptomatic AF patients referred for catheter ablation, findings can only be transferable to this population.

## 6. Conclusions

The unpredictable nature of AF influenced many life situations and resulted in disabling symptoms and impaired HRQOL. Patients reported that AF limited their activities of daily living and caused different kinds of worries. It led to a need to find strategies for managing daily living. The mixed methods design deepens our understanding of challenges faced by patients. It is vital to understand the extent to which patients can be affected and that the relationships between patients and their relatives can change. Healthcare providers need to be aware of supporting patients and relatives with relevant and updated information and to integrate them into the care.

### 6.1. Implications for Practice

- The level of symptoms and concerns relating to AF can vary between patients, and for many, their activities of daily living are limited substantially. Assessing and discussing symptoms and quality of life are important parts of follow-up to target and support high-risk patients.
- Patients with AF experience different dimensions of worries, and their strategies for management can be strengthened through an integrated care approach focusing on psychoeducational support.

## Figures and Tables

**Table 1:** Demographic and characteristics of patients.

Patient characteristics	Patients *N* = 19^1^	Median values
Age, years (25^th^–75^th^)		61 (54–69)
Sex, male	12	

*Marital status*		
Married/living with partner	14	
Single	5	

*Educational level*		
Primary school	2	
Upper secondary school	4	
High school	5	
University degree	8	

*Employment*		
Employed	12	
Unemployed	1	
Retired	6	

Years since AF diagnosis (range)		5 (1–31)

*Pattern of AF*		
Paroxysmal	14	
Persistent	5	

*Frequency of AF during the last 3 months*		
<5 occasions	4	
5–15 occasions	7	
16–30 occasions	3	
Persistent	5	

*Usual duration of AF episodes*		
1 to <7 hours	4	
7 to <24 hours	7	
24 hours to <2 days	1	
2 to 7 days	1	
>7 days	5	

*Comorbidities*		
Hypertension	8	
Diabetes mellitus	3	
Heart failure	4	
COPD	2	
Ischemic heart disease	2	
Stroke/subarachnoid haemorrhage	1/1	

*Treatment*		
Pacemaker/ICD	1/1	
Antiarrhythmic medication		
Class I	6	
Class II	14	
Class III	6	
Class IV	2	
Digoxin	0	
Anticoagulants	19	

AF = atrial fibrillation; ICD = implantable cardioverter defibrillator; COPD = chronic obstructive pulmonary disease. Median values are presented with 25th to 75th percentiles within brackets. Patients can have more than one antiarrhythmic drug. All patients on anticoagulants were treated with warfarin/Waran. ^1^One patient was interviewed only and did not complete the questionnaires.

**Table 2:** Results from the Arrhythmia-Specific Questionnaire in Tachycardia and Arrhythmia, the Hospital Anxiety and Depression Scale, the Control Attitudes Scale, and the Short-Form 36 items.

Questionnaires *N* = 18	Scale score, median values (25^th^–75^th^)
ASTA 9-item symptom scale	9.5 (6.8–14)
ASTA 13-item HRQOL scale	14.5 (10.8–21.5)
HADS anxiety scale	3.5 (1.8–8.3)
HADS depression scale	3.5 (2.0–7.5)
CAS helplessness scale	7.5 (5.3–10)
CAS perceived control scale	6.5 (4–8)
SF-36 8 scales	
Physical functioning	77.5 (60–91.3)
Role-physical	50 (0.0–81.3)
Bodily pain	84 (65.5–100)
General health	67 (37.5–72)
Vitality	47.5 (23.8–65)
Social functioning	81.3 (25–100)
Role-emotional	100 (33.3–100)
Mental health	82 (64–89)

Median values are presented with 25th to 75th percentiles within brackets. ASTA = Arrhythmia-Specific Questionnaire in Tachycardia and Arrhythmia. Each score in the ASTA symptom and the ASTA health-related quality of life (HRQOL) scales ranges between 0–27 and 0–39 respectively, where a higher score indicates higher symptom burden and/or a worse effect on HRQOL. SF-36 = Short-Form 36 items. The scores in the eight scales of the SF-36 range between 0 and 100, where 0 represents worst possible health and 100 the best possible health. HADS = Hospital Anxiety and Depression Scale. The two sections assess anxiety (HADS-A) and depression (HADS-D). The score for each subscale ranges from 0 to 21, with higher scores reflecting more psychological distress. CAS = Control Attitudes Scale. The scale consists of two items about perceived control and two about helplessness. Two items reflect patients' own perceptions and two reflect patients' perception about family members' perceived control. Scores range between 1 and 7, and the total scale scores range between 4 and 28, where higher scores indicate greater perceived control.

## Data Availability

The data used to support the findings of this study are available from the corresponding author upon request (interviews and questionnaires).

## Supplementary Materials

Supplementary Materials Table 1: demographic and characteristics of patients. Table 2: results from the Arrhythmia-Specific Questionnaire in Tachycardia and Arrhythmia, the Hospital Anxiety and Depression Scale, the Control Attitudes Scale, and the Short-Form 36 Items.
