# Supplementary material for: Patients' Experiences of Living with Atrial Fibrillation: A Mixed Methods Study
Source: Cardiol Res Pract. 2019 Dec 3;2019:6590358. doi: 10.1155/2019/6590358 (PMC6915031; doi:10.1155/2019/6590358)
Supplement: Supplementary Materials — Table 1: demographic and characteristics of patients. Table 2: results from the Arrhythmia-Specific Questionnaire in Tachycardia and Arrhythmia, the Hospital Anxiety and Depression Scale, the Control Attitudes Scale, and the Short-Form 36 Items. [file 6590358.f1.docx]

**Table 1** Demographic and Characteristics of Patients

| **Patient characteristics** | **Patients**  **N=19^1^** | **Mean values (±SD)** |
| --- | --- | --- |
| Age, years  (range) |  | 60 (±9.4)  (45-75) |
| Gender, Male | 12 |  |
| Marital Status:   - Married/living with partner - Single | 14  5 |  |
| Educational level:   - Primary school - Upper secondary school - High school - University degree | 2  4  5  8 |  |
| Employment:   - Employed - Unemployed - Retired | 12  1  6 |  |
| Years since AF diagnosis  (range) |  | 5 (1-31) |
| Pattern of AF   - Paroxysmal - Persistent | 14  5 |  |
| Frequency of AF during the last three months   - < 5 occasions - 5-15 occasions - 16-30 occasions - Persistent | 4  7  3  5 |  |
| Duration of AF episode:   - 1-7 hours - 7-24 hours - 2-7 days - > 7 days | 3  10  1  5 |  |
| Comorbidities:   - Hypertension - Diabetes mellitus - Heart failure - COPD - Ischemic Heart Disease - Stroke/subarachnoid haemorrhage | 8  3  4  2  2  1/1 |  |
| Treatment:  Pacemaker/ ICD:  Anti-arrhythmic medication:   - Class I - Class II - Class III - Class IV - Digoxin   Anticoagulants: | 1/1  6 14  6  2  0  19 |  |

AF= Atrial Fibrillation. ICD = Implantable Cardioverter Defibrillator, COPD=Chronic Obstructive Pulmonary Disease. Patients can have more than one anti-arrhythmic drug.

All patients on anticoagulants were treated with warfarin/Waran.

**^1^** One patient was only interviewed but did not complete the questionnaires.

**Table 2**. Results from the Arrhythmia-Specific questionnaire in Tachycardia and Arrhythmia, the Hospital Anxiety and Depression Scale, the Control Attitudes Scale and the Short-Form 36-Items

| **Questionnaires**  N=18 | **Scale score, mean values**  (±SD) |
| --- | --- |
| **ASTA** 9 items symptom scale | 37.2 (±16,1) |
| **ASTA** 13 items HRQOL scale | 43.9 (±21.9) |
|  |  |
| **HADS** anxiety scale | 5.0 (±4.5) |
| **HADS** depression scale | 5.3 (±4.2) |
|  |  |
| **CAS** scale | 14.3 (±5.4) |
|  |  |
| **SF-36 8 scales** | **Scale score, mean values**  (±SD) |
| Physical functioning | 73  (±22) |
| Role-physical | 42  (±40) |
| Bodily pain | 77  (±23) |
| General health | 58  (±21) |
| Vitality | 45  (±23) |
| Social functioning | 69  (±32) |
| Role-emotional | 70  (±38) |
| Mental health | 74  (±23) |

ASTA= Arrhythmia-Specific questionnaire in Tachycardia and Arrhythmia. Each score in the ASTA symptom and the ASTA Health-Related Quality of Life (HRQOL) scales ranges between 0-100, where a higher score implies higher symptom burden and/or a worse effect on HRQOL.

SF-36 =Short-Form 36 items. The scores in the SF-36 eight scales range between 0-100, where 0 represents worst possible health and 100 the best possible health.

HADS=Hospital Anxiety and Depression Scale. The two sections assesses anxiety (HADS-A) and depression (HADS-D). The score for each subscale ranges from 0 to 21, with higher scores reflecting more psychological distress.

CAS=Control Attitudes Scale. The scale consists of two items about perceived control and two about helplessness. Two items reflect the patient’s own perceptions and two reflect the patients’ perception about family members’ perceived control. The scoring ranges between 1- 7, and the total scale score ranges between 4 to 28, where higher scores indicate greater perceived control.
